# Molecular Pathways and Targeted Therapies for Malignant Ovarian Germ Cell Tumors and Sex Cord–Stromal Tumors: A Contemporary Review

**DOI:** 10.3390/cancers12061398

**Published:** 2020-05-29

**Authors:** Asaf Maoz, Koji Matsuo, Marcia A. Ciccone, Shinya Matsuzaki, Maximilian Klar, Lynda D. Roman, Anil K. Sood, David M. Gershenson

**Affiliations:** 1 Department of Medicine, Boston University School of Medicine and Boston Medical Center, Boston, MA 02118, USA; asaf.maoz@bmc.org; 2 Division of Gynecologic Oncology, Department of Obstetrics and Gynecology, University of Southern California, Los Angeles, CA 90033, USA; marcia.ciccone@med.usc.edu (M.A.C.); zacky_s@gyne.med.osaka-u.ac.jp (S.M.); lroman@med.usc.edu (L.D.R.); 3 Norris Comprehensive Cancer Center, University of Southern California, Los Angeles, CA 90033, USA; 4 Department of Obstetrics and Gynecology, University of Freiburg, 76100 Freiburg, Germany; maximilian.klar@uniklinik-freiburg.de; 5 Department of Gynecologic Oncology and Reproductive Medicine, The University of Texas MD Anderson Cancer Center, Houston, TX 77033, USA; asood@mdanderson.org (A.K.S.); DGershen@mdanderson.org (D.M.G.)

**Keywords:** non-epithelial ovarian tumors, malignant ovarian germ cell tumors, ovarian sex cord–stromal tumors, targeted therapy, precision medicine, cancer genomics

## Abstract

Non-epithelial ovarian tumors are heterogeneous and account for approximately 10% of ovarian malignancies. The most common subtypes of non-epithelial ovarian tumors arise from germ cells or sex cord and stromal cells of the gonads. These tumors are usually detected at an early stage, and management includes surgical staging and debulking. When indicated for advanced disease, most respond to chemotherapy; however, options for patients with refractory disease are limited, and regimens can be associated with significant toxicities, including permanent organ dysfunction, secondary malignancies, and death. Targeted therapies that potentially decrease chemotherapy-related adverse effects and improve outcomes for patients with chemotherapy-refractory disease are needed. Here, we review the molecular landscape of non-epithelial ovarian tumors for the purpose of informing rational clinical trial design. Recent genomic discoveries have uncovered recurring somatic alterations and germline mutations in subtypes of non-epithelial ovarian tumors. Though there is a paucity of efficacy data on targeted therapies, such as kinase inhibitors, antibody–drug conjugates, immunotherapy, and hormonal therapy, exceptional responses to some compounds have been reported. The rarity and complexity of non-epithelial ovarian tumors warrant collaboration and efficient clinical trial design, including high-quality molecular characterization, to guide future efforts.

## 1. Introduction

**Figure 1:**
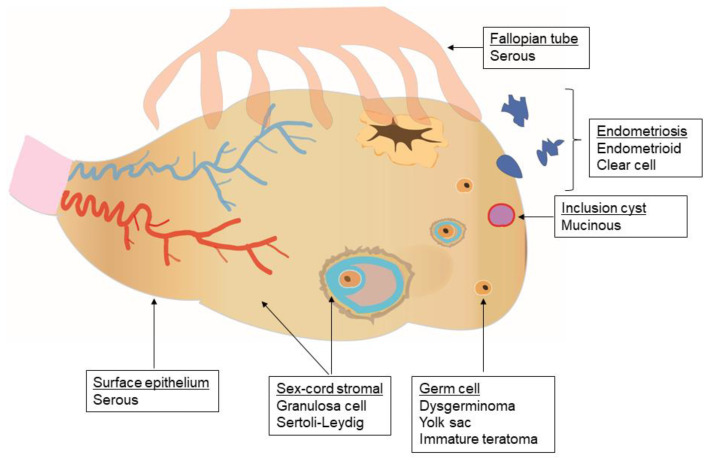
Epithelial and non-epithelial cells of the ovary. The cells from which the primary epithelial and non-epithelial ovarian tumors originate are depicted. Epithelial ovarian cancer arises from the surface epithelium of the ovary, fallopian tubes, and peritoneum. Non-epithelial ovarian tumors arise from gonadal germ cells, sex cord–stromal cells, and other non-epithelial cells.

Non-epithelial ovarian tumors are an uncommon group of malignancies that arise from germ cells, sex cord cells, and/or stromal cells of the ovary. The term non-epithelial is used to distinguish these tumors from their epithelial counterparts, which usually arise from the external lining of the ovaries or the fallopian tube epithelium (Figure 1). This histological distinction is based on the World Health Organization’s classification of ovarian tumors [1,2] and has important genomic, epigenetic, and clinical implications [3,4].

Non-epithelial ovarian tumors account for approximately 8–10% of ovarian malignancy cases [3,5,6], representing approximately 2200 new cases per year in the United States [3]. This group of tumors is heterogeneous and comprised of malignant ovarian germ cell tumors (MOGCT), malignant sex cord–stromal tumors (SCST), and other tumors. These categories are further subdivided into a multitude of histologically and clinically diverse groups. Compared with epithelial malignancies, these tumors disproportionally affect younger patients, with some types most frequently occurring in the pediatric population. The rarity and heterogeneity of non-epithelial ovarian tumors result in a paucity of high-quality data to guide clinical care of patients with these tumors.

This review will address potential molecular therapeutic approaches to non-epithelial ovarian tumors, focusing on germ cell tumors and sex cord–stromal tumors of the ovary. General considerations for the management of these tumors, including epidemiology, surgical staging, and treatment, and common chemotherapy regimens will be briefly covered.

## 2. General Principles

Non-epithelial ovarian tumors often occur at a younger age than epithelial ovarian cancer, with some tumors occurring predominantly in children, adolescents, or young adults [3,5]. Diagnostic and therapeutic considerations for these patients include potential preservation of fertility and preventing long-term toxicity of chemotherapy, including organ dysfunction and secondary cancers. Fortunately, as a group, non-epithelial ovarian tumors are usually detected at an early stage and are associated with a favorable prognosis [7,8].

Like epithelial ovarian cancer, staging of non-epithelial ovarian tumors is performed surgically and is crucial for informing prognosis, subsequent surveillance, and therapy [9,10,11]. A comprehensive review of the outcomes associated with specific staging procedures is outside the scope of this review [7,8,12]. Complete staging is recommended by most gynecologic oncology societies [9,13], but it is unclear how this practice affects survival [14]. For example, the typical surgical staging of children by pediatric surgeons is less comprehensive than that of gynecologic oncologists for adults [14]. Generally, complete staging may contribute to increased perioperative morbidity [15], while incomplete staging is associated with a higher risk of tumor recurrence [11,16,17]. For patients who have given birth or are postmenopausal, surgical staging includes bilateral salpingo-oophorectomy and total abdominal hysterectomy. However, fertility-sparing surgery with unilateral salpingo-oophorectomy is often pursued for younger patients, preserving the uterus and the contralateral ovary [15,18,19,20,21,22]. For patients with bilateral tumors, cystectomy of one ovary can be considered to preserve fertility [13].

Peritoneal fluid is typically sampled. Cytoreduction of the visible tumor, random biopsies of the peritoneal surfaces and other organs, and omentectomy are also recommended, although not infrequently omitted [14,17]. Routine lymphadenectomy is controversial and depends on the tumor subtype and pre- and intra-operative findings [15,23,24]. Patients with adult granulosa cell tumors in particular have low rates of lymph node metastases and do not seem to benefit from lymphadenectomy [25,26,27,28]. In addition to obtaining diagnostic and prognostic information, surgery is a major component of therapy. Early stage tumors can be cured with the surgical procedure alone and may not require adjuvant systemic treatment [7,8,29].

Patients with advanced disease typically require treatment with platinum-based chemotherapy regimens [13]. However, these regimens are associated with significant toxicity, including long-term organ dysfunction, secondary malignancy, and death.

In the setting of platinum resistance, patients are encouraged to enroll in clinical trials due to limited and understudied therapeutic options [30,31]. Several second-line chemotherapeutic regimens have been proposed, including high dose chemotherapy with an autologous stem cell transplant [32,33]. But while effective for some patients, high-dose chemotherapy is highly toxic, with a 6% death rate in one of the aforementioned trials [32].

Hence, incorporating effective targeted treatments for non-epithelial ovarian tumors could be beneficial for reducing long-term toxicity from chemotherapy as well as for addressing the unmet needs in the recurrent or refractory setting. Recent characterization of molecular and genetic aberrations of non-epithelial ovarian tumors has uncovered new potential therapeutic targets that differ by the tumor cell of origin and subtype.

## 3. Malignant Ovarian Germ Cell Tumors (MOGCTs)

The most common group of non-epithelial ovarian tumors is malignant ovarian germ cell tumors (MOGCTs). In children, they represent the majority (75%) of malignant ovarian tumors [34]. MOGCTs are infrequent in older patients and occur predominantly in adolescents and young adults [34]. The most common types of MOGCTs (Table 1) are dysgerminoma and immature teratoma which comprise 65–70% of MOGCTs, followed by yolk sac tumors and mixed germ cell tumors [35,36]. These tumors reflect the pluripotent potential of primordial germ cells to differentiate into all somatic (endoderm, mesoderm, ectoderm) and extra-embryonic tissues [37]. MOGCTs can lead to elevations of tumor markers in the peripheral blood, including alpha-fetoprotein (AFP), beta-human chorionic gonadotropin (hCG) and lactate dehydrogenase (LDH) [7,38,39].

MOGCTs represent approximately 3% of all ovarian tumors in the United States, with 4 cases per 1,000,000 women [3]. The incidence of MOGCTs is estimated to be slightly higher among Asian, Hispanic, and non-Hispanic black women than among non-Hispanic white and American Indian/Native Alaskan women [3]. The proportion of ovarian malignancies attributed to MOGCTs also varies across and within different geographic regions worldwide, with the highest proportion reported in East Asia and Central America [5,34]. The concordance of international variation with ethnicity in the United States raises the possibility of genetic susceptibility to MOGCTs, but further studies are needed to evaluate this hypothesis.

MOGCTs are often diagnosed at an early stage. In the United States, approximately 69% of patients with available data are diagnosed at Stage I [3]. Five-year cause-specific survival for these patients is 99% across all races. Approximately 26.5% are diagnosed at Stage II–III, both with five-year cause-specific survival rates of over 90% with modern therapy. Less than 5% of patients with available stage data are diagnosed at Stage IV, with a 69% five-year survival [3].

For early stage dysgerminoma and immature teratoma, surgery without adjuvant chemotherapy is currently recommended [13]. The reason is threefold: (i) outcomes with surgery alone are often curative; (ii) in the setting of recurrent or residual disease, responses to chemotherapy are excellent; and (iii) this approach best preserves ovarian reserve and fertility [43,44].

For those who require adjuvant therapy, the most common first-line chemotherapy regimen consists of bleomycin, etoposide, and cisplatin (BEP). This regimen is curative for most patients with limited or no residual disease after surgery. For those with bulky residual disease, 50–60% achieve cure with adjuvant chemotherapy [45]. While very effective, this regimen may result in significant short- and long-term toxicities [46,47,48]. Furthermore, a substantial proportion of patients remain disease-free without adjuvant chemotherapy [46], and therapies for disease recurrence are often curative. These facts call into question the benefit of adjuvant treatment. Adverse effects of BEP occur in up to 30% of patients, including cisplatin-induced peripheral neuropathy, ototoxicity, and nephrotoxicity, potentially fatal secondary malignancies, bleomycin-associated lung injury, bone marrow suppression, and cardiomyopathy [46,49]. Most patients who receive BEP, however, retain ovarian function and can achieve pregnancy [50,51,52]. Given the toxicity of BEP, less toxic regimens have been proposed, including carboplatin and etoposide [50,53], but these remain investigational. Despite encouraging retrospective data, results from testicular germ cell tumors raise concerns that carboplatin is less effective than cisplatin for certain non-epithelial ovarian tumors [54].

As is the case of all ovarian cancers, chemotherapy for recurrent diseases tends to be more effective in those with platinum-sensitive disease, with remission rates on the order of >60% with salvage platinum-based chemotherapy, but only <30% experiencing long-term survival with platinum-refractory disease. Active agents include platinum agents, vinblastine, ifosfamide, taxanes, and gemcitabine [45]. There is controversy regarding the need for adjuvant chemotherapy for early-stage, non-dysgerminoma MOGCTs, including stage IA grade 2–3 immature teratoma, stage IA or IB yolk sac tumors, and other less common histologies [14,55]. Observation alone is often proposed for these patients by pediatric oncology and European gynecologic oncology societies, whereas adjuvant chemotherapy has typically been offered by gynecologic oncologists in the United States. Protocol AGCT 1531 (NCT03067181) evaluates the risks and benefits of these approaches.

**Figure 2:**
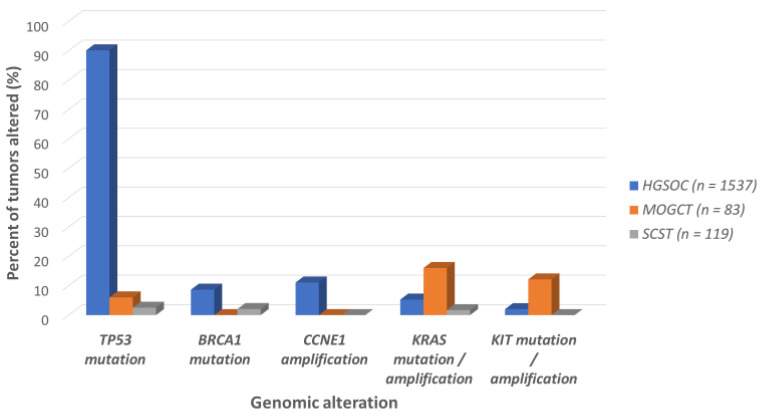
Differences in genetic alterations between epithelial and non-epithelial ovarian tumors (GENIE/AACR database). Aggregated data for 5 genes were derived from the GENIE/AACR database for high-grade serous ovarian cancer (HGSOC) (an epithelial ovarian cancer), female germ cells tumors (MOGCT) and female sex cord–stromal tumors (SCST). Alterations included mutations (excluding synonymous mutations), amplifications, homozygous deletions, and fusions. Alterations with <1% frequency across the three tumor categories were excluded. The breakdown of tumor subtypes in this database does not reflect their prevalence in the population.

The genomics of MOGCTs are understudied. Available data suggest that MOGCTs have a low mutational burden with marked aneuploidy [42]. This pattern is hypothesized to arise from abnormal segregation of chromosomes during meiosis and/or mitosis [42,56]. A whole exome sequencing study of 24 MOGCTs found a median of 2.5 (range 0–8) non-synonymous mutations per tumor; an average of 35% of the genome was affected by copy number alterations in 87 patients. The most common copy number alteration was gain of chromosome 12p, containing the oncogene *KRAS* [42]. This alteration was found in 82% of dysgerminomas, 58% of yolk sac tumors, and 43% of mixed germ cell tumors, but not in immature teratomas. In contrast to epithelial ovarian cancer [57], *TP53* mutations were not detected in MOGCTs; the most common mutations were in the genes *KIT* and *KRAS* [42], akin to testicular germ cell tumors [58]. Similar differences in mutations between epithelial and non-epithelial ovarian tumors can be seen in the Genomics Evidence Neoplasia Information Exchange of the American Association for Cancer Research (GENIE/AACR, version 7.0) database (Figure 2) [59,60]. Importantly, any genomic analysis grouping all MOGCTs and/or SCSTs is limited by the vast heterogeneity of tumors within each group.

The immune response to MOGCTs is understudied. Early evidence suggested that similar to testicular germ cell tumors, MOGCTs (primarily, dysgerminomas) are characterized by immune infiltrates that may have a prognostic value [61,62,63,64,65]. These infiltrates are comprised of several cell types, including T and B lymphocytes, that can organize as tertiary lymphoid structures with germinal center-like structures [66]. Tertiary lymphoid structures are found within MOGCTs, whereas they are commonly peritumoral in other cancers [67,68,69]. Limited data from testicular tumors suggest that tumor progression is accompanied by a decrease in T cells and natural killer cells and an increase in regulatory T cells and macrophages [70]. Programmed death ligand-1 (PD-L1) expression is also common in male germ cell tumors [71].

### 3.1. Dysgerminoma

Dysgerminoma is the most common MOGCT, accounting for 30–35% of cases. Dysgerminoma histologically resembles testicular seminoma, with correspondingly similar immunohistochemistry and chemosensitivity [30,72]. Though bilateral disease is present in approximately 10–15% of cases, fertility-sparing surgery can still be considered given its high chemosensitivity [13]. Adjuvant chemotherapy is not typically administered for stage IA disease and is controversial for patients with bilateral disease, ovarian capsule rupture, and positive peritoneal/ascitic cytology (stages IB–IC) [13]. Adjuvant BEP is typically recommended for patients with stage II–IV disease [13]. Carboplatin and etoposide is another potential regimen that can be used in these patients as discussed above [53].

A minority of patients with dysgerminoma have gonadal dysgenesis. However, the karyotypic abnormalities involved in gonadal dysgenesis, including the presence of Y chromosome material, are a significant risk factor for the development of dysgerminoma and concurrent gonadoblastoma (a rare neoplasm containing both germ cell and sex cord–stromal cells) [50,73,74,75]. Therefore, bilateral oophorectomy is typically recommended for patients with Y chromosome material.

**Figure 3:**
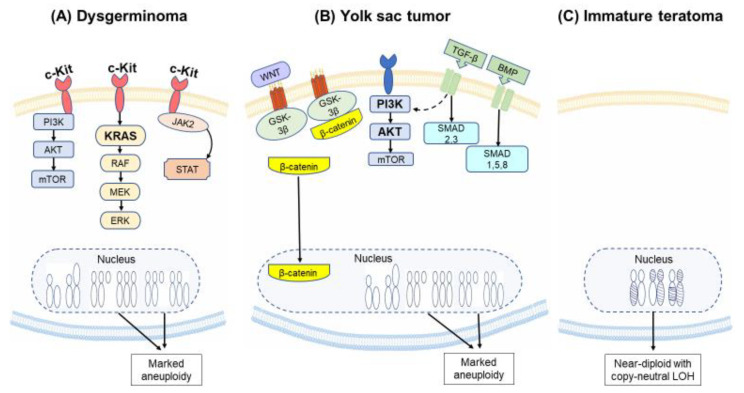
Common alterations in malignant ovarian germ cell tumors. Common alterations in the most prevalent malignant ovarian germ cell tumors (MOGCTs) are shown. (**A**) Dysgerminomas frequently demonstrate mutations in c-*KIT* and *KRAS*. (**B**) Yolk sac tumors have frequent amplifications of the genes *PIK3CA* and *AKT1* in the PI3K/AKT/mTOR pathway. Both dysgerminomas and yolk sac tumors are characterized by marked aneuploidy, whereas (**C**) immature teratoma is characterized by near-diploid copy neutral loss of heterozygosity (LOH).

*KIT* mutations and amplifications have been described in 30–50% of dysgerminomas [42,59,76]. *KIT* is a tyrosine kinase receptor that can lead to tissue-specific activation of several intracellular signaling pathways, including the RAS-RAF-MEK-ERK/JNK, PI3K-AKT-mTOR, and JAK/STAT pathways [77] (Figure 3A). *KIT* mutations are common in gastrointestinal stromal tumors (GIST), where they are also predictive of response to the tyrosine kinase inhibitor, imatinib [78].

Chromosomal gains of the 12p arm containing *KRAS*, a gene commonly found in testicular germ cell tumors [37], occur in up to 80% of patients with dysgerminoma [42,79]. *KRAS* is an oncogene implicated in the pathogenesis of multiple cancers and is a major driver of the RAS-RAF-MEK-ERK/JNK pathway. While the direct targeting of KRAS is challenging [80], encouraging results have been reported with the MEK1/2 inhibitor trametinib in low-grade serous ovarian cancer, an uncommon epithelial ovarian cancer subtype [81].

Overexpression of several genes has been described in dysgerminomas, including *CASP8, CDH3, CXCL10*, and *IL6R* and the pluripotency-related genes *NANOG, POU5F1, POU5F1B, PLBD1*, and *PDPN* [37]. Significant limitations of these analyses include the grouping of dysgerminoma with testicular seminoma and the lack of normal tissues to include as controls.

### 3.2. Yolk Sac Tumors

Yolk sac tumors account for approximately 15% of MOGCTs [36]. Yolk sac histology is typically associated with elevated AFP and is considered an adverse prognostic factor [7,13,36,82]. Chemotherapy has traditionally been offered even for Stage IA disease, although the European guidelines suggest it is optional to forego adjuvant chemotherapy in this setting [13,83]. More advanced stages warrant adjuvant chemotherapy. BEP is the recommended regimen [13,83].

Yolk sac tumors are aneuploid with characteristic copy number alterations, including chromosome 12p gain in approximately 60% of the tumors, but are not associated with specific recurrent mutations [37,42]. Alterations in the PI3K/AKT/mTOR signaling pathway (Figure 3B), which occur frequently in certain subtypes of epithelial ovarian cancers [81,84,85], seem to be enriched in tumors with a yolk sac component (72%). *PIK3CA* and *AKT1* were amplified in 42% and 37% of tumors with a Yolk sac component, respectively [42]. Targeting this pathway in epithelial ovarian cancer has yet to significantly improve outcomes [81,84]; similar challenges may arise with yolk sac tumors. Gene expression studies suggest that TGF-β/BMP and Wnt/β-catenin signaling pathways are activated in yolk sac tumors, but not in dysgerminomas [41]. TGF-β/BMP is involved in embryonic development and possibly MOGCT development [41]. The Wnt/β-catenin is activated in many cancer types, and inhibitors of this pathway are in clinical trials [86].

### 3.3. Immature Teratoma

Immature teratoma, sometimes referred to as malignant teratoma, has comparable or higher incidence than dysgerminoma (30–35% of MOGCTs) and may also present with bilateral disease [36,87]. It is thought to be chemo-resistant and surgical management is often needed for recurrent or advanced disease [46,55]. However, adjuvant BEP is recommended for advanced stage disease and is controversial for Stage IA disease with histological grade 2–3 [46,55]. Long-term overall survival is slightly worse than in patients with dysgerminoma (84% vs. 89.1% overall survival with a median follow-up of 126 months) [36].

Unlike other MOGCTs, immature teratoma is typically diploid [37,42], and chromosome 12p gain and *KIT/KRAS* mutations are uncommon [42]. Whole exome sequencing from 10 patients, most with advanced disease, uncovered extensive loss of heterozygosity without recurring somatic mutations. Variants without known functional significance were detected in *TP53, NF1, CTNBB1*, and *NOTCH2* (once each). The authors show that copy neutral loss of heterozygosity results from meiotic errors at different stages that can be identified based on the copy number variation profile of individual tumors (Figure 3C) [56]. This study also suggested that while bilateral disease arises from different clonal events, spread to the peritoneum is driven by a single clone, even in bilateral disease [56]. Seventeen patients with immature teratoma are included in the GENIE/AACR database. While recurrent mutations were not common, variants of unknown significance in *POLE, BRCA2* and *ATM* (one each) were detected.

### 3.4. Mixed Germ Cell Ovarian Tumors and Others

Mixed MOGCTs contain more than one histological form and account for approximately 5% of the MOGCTs. Embryonal carcinoma, choriocarcinoma, and polyembryoma cell types account for 5–10% of MOGCTs and have the worst prognosis. They rarely exist in pure form [36]. Adjuvant BEP is typically recommended for these histologies at all stages [13,83]. Their genomic landscape is thought to be determined by individual tumor components [37,42]. Embryonal carcinoma expresses CD30 in approximately 80% of cases, although expression may decrease after treatment with chemotherapy [88,89,90].

## 4. Malignant Sex Cord–Stromal Tumors (SCSTs)

Malignant sex cord–stromal tumors (SCSTs) arise from the primitive sex cord and/or stromal cells of the gonads (Figure 1), including granulosa, theca, Sertoli, or Leydig cells, as well as fibroblasts. SCSTs are rare; middle-aged women are the most commonly affected [5,6]. In the United States, they represent approximately 2% of ovarian malignancies, with 3 cases per 1,000,000 women. SCSTs appear to be most common among non-Hispanic black women and least common among Asian/Pacific Islander women in the United States [3], though the existing epidemiologic data are substantially limited [6]. Though commonly diagnosed at an early stage, five-year cause-specific survival is slightly lower than for MOGCTs, at 88% across all the stages uniformly. In the United States, Stage I, II, III, and IV disease at diagnosis was found in 69%, 12%, 14%, and 5% of the cases with available data, respectively [3]. The corresponding five-year cause-specific survival rates were 98%, 84%, 61%, and 41%.

When indicated, common chemotherapy regimens for SCSTs include BEP, cisplatin and etoposide (EP), and carboplatin with paclitaxel [13]. The latter is increasingly used in clinical practice. A phase II randomized trial is underway comparing carboplatin and paclitaxel to BEP for sex cord–stromal cell tumors (NCT01042522). Response to taxanes in incompletely resected recurrent disease has been measured at 42% [91].

The most common subtypes of SCSTs (Table 1) are granulosa cell tumors, accounting for over 70% of SCSTs in most series [87,92,93]. Sertoli–Leydig tumors are the next most frequent group. SCSTs often secrete hormones, including inhibin, estradiol, testosterone, and anti-Müllerian hormone, which can be measured and followed as tumor markers. These can lead to such symptoms as virilization, menstrual changes, post-menopausal bleeding, and precocious puberty [6]. SCSTs, unlike MOGCTs, are not characterized by widespread genomic instability with copy number variations [94], although recurrent chromosomal abnormalities have also been described [40].

### 4.1. Granulosa Cell Tumors

The adult and juvenile granulosa cell histological subtypes comprise the majority (>70%) of SCSTs in the adult and children/adolescent age groups, respectively [6,87,92,93]. Overall, adult granulosa cell tumors are much more common [95]. Granulosa cell tumors often secrete estradiol, which induces proliferation of the endometrium. Endometrial hyperplasia and endometrial cancer, which is associated with granulosa cell tumors, can manifest as abnormal uterine bleeding [96,97]. Surgery is the mainstay of treatment for early stage disease, commonly followed by platinum-based adjuvant chemotherapy for metastatic disease. The two most common regimens are BEP and carboplatin with paclitaxel [6,13].

**Figure 4:**
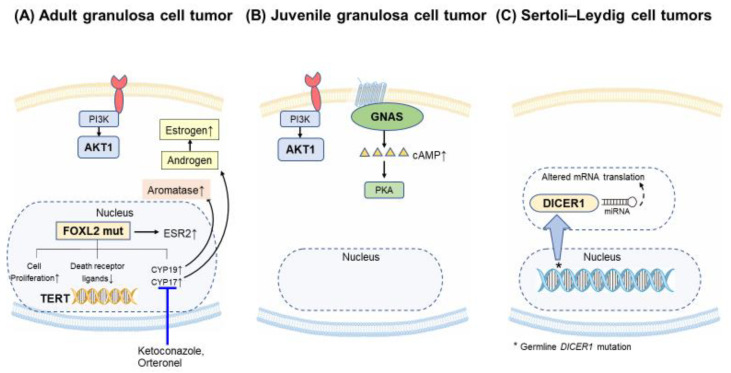
Common alterations in sex cord–stromal cell tumors. (**A**) Adult granulosa cell tumors almost ubiquitously have a somatic mutation in *FOXL2*, leading to transcriptional alterations, including in cytochrome P450 (CYP) 17 and 19 expression. (**B**) Juvenile granulosa cell tumors have mutations in *GNAS* in approximately 30% of cases. *AKT1* is the most commonly amplified gene. (**C**) Approximately 60% of Sertoli–Leydig tumors are associated with a mutation in the ribonuclease III (RNAse III) DICER1, which can be a germline mutation predisposing to several cancers. Mut—mutated.

Genomic studies of SCSTs demonstrate that a single somatic mutation in *FOXL2* (C134W) is almost ubiquitous in adult granulosa cell tumors, occurring in up to 97% of cases [94,98]. Since some adult granulosa cell tumors can be difficult to definitively diagnose based on histology and immunohistochemistry alone, *FOXL2* has been suggested for the molecular diagnosis of these tumors [95,99,100]. The presence of the *FOXL2* mutation in tumors with equivocal histological diagnosis may aid in the classification of the tumor as an adult granulosa cell one [100]. Conversely, the tumors classified as the adult granulosa cell ones, but lacking the characteristic *FOXL2* mutation, may represent a histological misclassification [101]. FOXL2 is a transcription factor that is involved in regulation of hormone production, cell cycle, and apoptosis [102,103]. The precise mechanism by which this mutation promotes tumor formation is unclear; FOXL2 possibly serves as a tumor suppressor [40,104], but others have postulated that it acts as an oncogene [105]. The somatic mutation may lead to dysregulation of multiple cellular processes. FOXL2 normally downregulates cytochrome P450 (CYP) 17, and the altered product may lead to an increase in CYP17, with resulting increased estrogen production (Figure 4A) [106]. In addition, the mutated *FOXL2* increases expression of CYP19/aromatase [107]. The *FOXL2* mutation was also detected in 50% of granulosa theca cell tumors, but it is uncommon in juvenile granulosa cell tumors. *FOXL2* is rarely mutated in other cancers, with mutations occurring in approximately 1% of all cancers profiled by the GENIE/AACR project and less than 5% of any individual tumor type apart from SCSTs [59,60]. Fewer than 10% of these *FOXL2* mutations (33/408) are the recurrent C134W mutation, and of the 33 C134W mutations, 31 (94%) were found in SCSTs [59,60]. The functional significance of *FOXL2* mutations in other cancers is outside the scope of this review.

*TERT* mutations are also common in adult granulosa cell tumors [108,109]. A specific mutation in the *TERT* promoter (*TERT* c.-124C>T), found in up to 40% of cases, was associated with more aggressive disease and worse overall survival [108,109].

Whole genome sequencing of ten granulosa cell tumors revealed no mutations in *BRCA1/2* and only a few mutations (10%) in the following genes: *TP53*, *PIK3CA, CTNNB1,* and *PIK3R1* [110]. These are all rare (<5%) in 86 predominantly adult granulosa cell tumors with the data available on the GENIE/AACR. Recurrent alterations in *KMT2D* can be found in over 20% of patients in this database [59,60]. Other recurrent alterations are found in a limited number of patients and require confirmation in larger cohorts.

Most granulosa cell tumors are diploid, but recurrent chromosomal copy number alterations, including trisomy 12, 14 and monosomy 22 have been described [108,111,112]. In a small series of ten patients that requires confirmation in larger cohorts, *AKT1* was the most commonly amplified gene [112], potentially leading to aberrations in the PI3K/AKT/mTOR pathway. In juvenile granulosa cell tumors, approximately 30% harbored a mutation in *GNAS* [113] in a cohort of thirty patients. In a small study of 16 patients, over 60% harbored a duplication of *AKT1* [114] (Figure 4B). *GNAS* encodes a subunit of G protein-coupled receptors that are bound by follicle-stimulating hormone (FSH) on the surface of granulosa cells and stimulate adenylyl cyclase activity, increasing production of cyclic AMP. Protein kinase A is thought to be the initial protein kinase activated by cyclic AMP and one of the most important mediators of cyclic AMP signal transduction [115]. Granulosa cell tumors also frequently express vascular endothelial growth factor (VEGF) [116,117,118] and platelet-derived growth factor (PDGFR) [119]. The rare hereditary syndromes, Ollier disease and Maffucci syndrome, are associated with increased risk of juvenile granulosa cell tumors [6].

### 4.2. Sertoli–Leydig Cell Tumors

Sertoli and Leydig cells are found in the normal testis. Sertoli–Leydig cell tumors are typically detected at an early stage and are often accompanied by androgen production; AFP elevations have been described [95]. If adjuvant chemotherapy is indicated, BEP or carboplatin and paclitaxel are typically recommended [13].

*DICER1* mutations have been described in approximately 60% of Sertoli–Leydig tumors [120] (Figure 4C). The prevalence of *DICER1* mutations in Sertoli–Leydig cell tumors may be even higher when accounting for potential histological misclassification [121]. In one series, they were not found in well-differentiated Sertoli–Leydig tumors, but were found in all moderately poorly differentiated tumors [121]. DICER1 is a member of the ribonuclease III (RNAse III) family involved in transcriptional regulation via miRNA (microRNA) modulation. Some cases with somatic *DICER1* mutations were also found to harbor a germline mutation in *DICER1*, predisposing to additional tumors including pleuropulmonary blastoma [122]. Thus, germline testing for *DICER1* should be offered to patients with these tumors. *DICER1* is rarely mutated in cancers that are not associated with germline *DICER1* mutations [59]. *FOXL2* mutations have been described in 10–20% of Sertoli–Leydig tumors [99,123]. One study found that *DICER1* and *FOXL2* mutations are mutually exclusive in Sertoli–Leydig tumors and that each mutation was associated with distinct clinicopathological features [99]. These data suggest that the molecular classification of Sertoli–Leydig cell tumors may be clinically relevant, but prospective trials are needed to evaluate this hypothesis.

### 4.3. Other SCSTs

Peutz–Jeghers syndrome caused by mutations in the serine-threonine kinase 11 gene (*STK11)* can predispose to a specific subtype of SCSTs. These SCSTs are classified as SCSTs with annular tubules containing tubules of Sertoli cells arranged around one or more hyaline bodies [6]. When arising in Peutz-Jeghers syndrome, they are thought to be benign, but outside of this syndrome, a malignant clinical course has been described [6]. Approximately 40% of patients can be associated with Peutz-Jeghers syndrome [124]. Malignant thecomas and fibrosarcomas are very rare.

## 5. Application of Targeted Therapies in Clinical Trials

**Figure 5:**
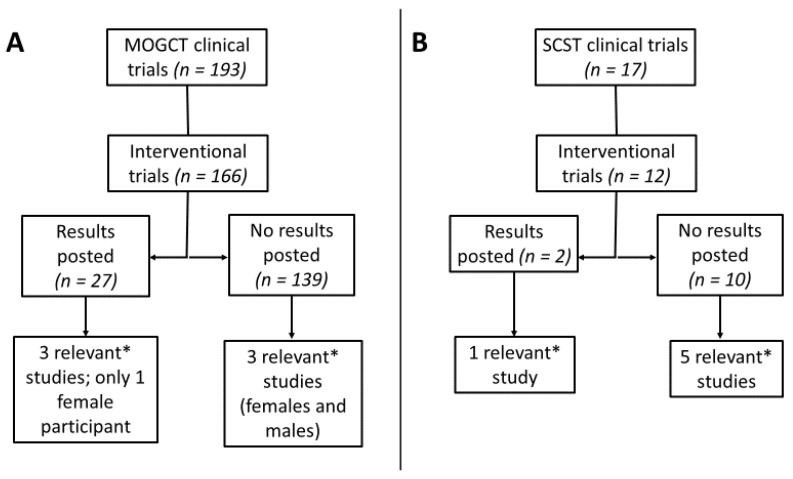
Clinical trials of targeted therapies of non-epithelial malignant ovarian tumors. Clinicaltrials.gov was searched for terms related to (**A**) malignant ovarian germ cells tumors (MOGCTs) and (**B**) sex cord–stromal tumors (SCSTs). Observational trials were not reviewed. * Trials evaluating drugs in multiple cancers, epithelial ovarian cancer and trials with chemotherapy-only interventions were classified as irrelevant. Relevant trials with results were evaluated for the number of female participants with non-epithelial ovarian tumors.

The rarity of non-epithelial ovarian tumors limits the ability to develop targeted therapies and evaluate them in well-powered clinical trials. A recent search of clinicaltrials.gov (Figure 5) revealed 166 interventional trials involving MOGCTs and only 12 involving SCSTs. Of the MOGCT trials, only 27 had results posted, and only 3 of these were specific to targeted therapy of MOGCTs. Within this group, there was only one female participant. For SCSTs, there was only one trial of targeted therapies with posted results. Several agents have been evaluated and trials from male testicular cancer patients may inform therapeutic strategies for non-epithelial ovarian tumors.

### 5.1. Tyrosine Kinase and Other Small Molecule Inhibitors

The alterations in signaling pathways discussed above and pan-cancer observations of kinase alterations [125] provide the rationale for using kinase inhibitors for the treatment of non-epithelial ovarian cancers. Imatinib, an inhibitor of several kinases, including c-KIT, has been evaluated in a phase 2 clinical trial including testicular and ovarian germ cells tumors (NCT00042952), but results of this trial are not currently available (Table 2). Rationale for this trial can be found in the frequent alterations in *KIT* in MOGCTs noted above. Imatinib has been reported to elicit a response in two anecdotal cases of granulosa cell tumors [126,127], with and without overexpression of c-KIT. Benefit, lack thereof, or harm cannot be established based on these limited data. A trial evaluating sunitinib, a multikinase inhibitor, for the treatment of germ cell tumors did not recruit any women (NCT00453310) [128]. Another trial evaluated oxaliplatin in combination with the cyclin-dependent kinase 9 (CDK9) inhibitor, alvocidib. This trial included only one female, and the results are available in the abstract form only. The trial did not meet its primary endpoint (NCT00957905). A myriad of other inhibitors have been proposed as being mechanistically relevant for the treatment of MOGCTs or SCSTs [129], including epidermal growth factor receptor (EGFR), PDGFR, insulin-like growth factor 1 (IGFR1) and VEGF. However, clinical data for their efficacy is lacking, and case reports are prone to publication bias.

### 5.2. Angiogenesis Inhibition

VEGF inhibition with bevacizumab monotherapy was evaluated in 36 patients with recurrent SCSTs [130]. The majority of them had granulosa cell tumors, and 92% had received prior chemotherapy. A 17% partial response rate accompanied by decreases in tumor markers met the pre-specified criteria for further investigation with combination regimens based on the prior retrospective data suggesting higher response rates in granulosa cell tumors treated with dual bevacizumab therapy and chemotherapy [131]. We anticipate full results of a bevacizumab/paclitaxel combination in this population (NCT01770301). Results in the abstract form suggest that the addition of bevacizumab does not improve the PFS; it was reported to lead to better response rates, but higher rates of adverse events [132].

### 5.3. Immunotherapy

Little is known about the immune response to non-epithelial ovarian tumors. The immune response to germ cell tumors is discussed above. Putative predictors of response to immunotherapy include tumor mutational burden, microsatellite instability, programmed death-1 (PD-1) and PD-L1 expression, and the presence of a host immune response within or around the tumor core [133,134]. Expression of the PD-L1 has been reported, in the abstract form only, in 75–80% of SCSTs [135], but immunotherapy has not been reported in a clinical trial of these tumors. Non-epithelial ovarian tumors are not characterized by a high mutational burden as discussed above, and microsatellite instability has not been reported in these tumors. Tumor lymphocyte infiltration occurs in germ cell tumors, but the trial of pembrolizumab for refractory germ cell tumors achieved no responses and did not include female participants (Table 2) [136]. Another trial of the dual checkpoint blockade with durvalumab, a PD-L1 inhibitor, and tremelimumab, a cytotoxic T lymphocyte-associated protein 4 (CTLA-4) inhibitor, is ongoing (NCT03158064). Other strategies of immunotherapy may become relevant for non-epithelial ovarian cancers. For example, recurrent clonal mutations such as those found in SCSTs can be targeted using adoptive T cell therapy [137], but the feasibility of this approach remains to be demonstrated and is dependent on HLA compatibility and efficient presentation to T cells. Cellular therapies are rapidly developing, and additional research is necessary to identify additional membrane-bound targets in non-epithelial ovarian cancer [138].

### 5.4. Endocrine Therapy

Endocrine therapy has been suggested primarily for granulosa cell tumors [139]. Recent discoveries about the hormonal effects of mutated *FOXL2* (Figure 4), which functions as a transcription factor that plays a role in granulosa cell development and in expression of hypophyseal gonadotropin-releasing hormone (GnRH) receptor expression [94], and the physiologic presence of follicle-stimulating hormone (FSH) receptors on granulosa cells provide the rationale for this approach. Promising reports are limited to case reports and series, while small trials have proven somewhat disappointing. Prior studies suggest efficacy using hormone blockade with leuprolide, a GnRH analog [140], and aromatase inhibition [141]. In one small but promising trial of 6 granulosa cell tumor patients treated with leuprolide, two patients experienced partial responses and 3—disease stability [140].

Several meta-analyses have also looked at hormonal therapy in granulosa cell tumors, including one that included 19 studies (31 patients) where patients received a variety of therapies, including aromatase inhibitors (AI) and tamoxifen, found an objective response rate of 71.0% with 25.8% complete responses. In this series, all responses (9/9) were to AI, and there were no responses to tamoxifen. Interestingly, of the 9 patients treated with AI, none had progressed at the time of publication, with the follow-up time ranging from 6–54 months after starting the treatment [142]. A more recent review in 2018 provided an updated meta-analysis including 12 different combinations of non-AI hormonal therapies (including GnRH agonists/antagonists, tamoxifen, progesterone, and diethylstilbestrol—DES), among 50 patients. The pooled analysis found clinical benefit for 33 of 50 patients and at least partial response in 17 patients [143]. The same review also compiled data for AI specifically, including letrozole, anastrozole, and exemestane. In this data set of 25 patients, 7 experienced complete response, 5—partial response, and 7—disease stability for a total of 19 patients with clinical benefit. Given the favorable side effect profile, the authors concluded that AI may be an alternative to chemotherapy [143].

There has been considerable interest surrounding AI given the data above and that FOXL2 is also known to activate aromatase [143]. The phase 2 PARAGON trial, which investigated anastrozole in a variety of gynecologic cancers, included a cohort of 41 postmenopausal recurrent granulosa cell tumor patients with estrogen receptor-positive disease. Results for this cohort, though somewhat disappointing compared with the above meta-analyses, have been published in the abstract form, showing a 9.8% response rate with 59% progression-free survival at 6 months [144].

A trial is evaluating the androgen receptor signaling inhibitor, enzalutamide, in these patients (NCT03464201), and a trial of the progesterone antagonist onapristone for patients with progesterone receptor-positive low-grade ovarian tumors, including granulosa cell tumors, is currently recruiting (NCT03909152).

Cytochrome P17 (CYP17) converts 17-hydroxyprogesterone to androstenedione and is downregulated by FOXL2. Mutations in *FOXL2* as noted in granulosa cell tumors, therefore, may result in increased androstenedione levels. Trials of cytochrome P450 (CYP) 17 inhibition with the nonsteroidal inhibitor orteronel and the anti-fungal ketoconazole for the treatment of granulosa cell tumors have been reported, but are yet to be published in peer-reviewed journals. A case report of ketoconazole for this indication suggested activity in a patient with multiple recurrences who experienced at least 10 months of disease stability following the regimen [145], leading to a clinical trial by the same group. Based on this single-arm trial of ketoconazole in six patients with adult granulosa cell tumor, only three of whom were confirmed to have the somatic *FOXL2* mutation, ketoconazole achieved stable disease in five patients and was granted an orphan designation for this indication by the European Medicines Agency. This data is available in the preprint form only [146]. Ortenerol was evaluated in ten patients in a trial that was terminated early due to slow recruitment [147]. The data available in the abstract form report a clinical benefit rate of 50%, with 3 patients achieving stable disease for over 12 months.

### 5.5. Other Agents

Non-epithelial ovarian tumors are not considered to harbor homologous recombination deficiency (HRD); mutations in *BRCA1/2* and other HRD-associated genes are uncommon (Figure 2). Poly (adenosine diphosphateribose) polymerase (PARP) inhibitors, which can confer synthetic lethality to cancer cells with HRD, have not been reported in clinical trials for non-epithelial ovarian tumors. Therapeutic compounds that may confer synthetic lethality to tumor cells with aneuploidy, such as MOGCTs, have also not been trialed [148] in these patients. The hypomethylating agent guadecitabine is being evaluated in combination with cisplatin for refractory germ cell tumors (NCT02429466), with signs of efficacy including 2/14 patients experiencing complete response. Only one patient in this trial had a MOGCT, which has only been reported in the abstract form. The data regarding the efficacy of serine-threonine kinase inhibitors for the treatment of non-epithelial tumors is also lacking, despite the data above suggesting pathway alterations in RAS-RAF-MEK-ERK/JNK and PI3K-AKT-mTOR.

### 5.6. Drugs Evaluated in Testicular Tumor

The histological and molecular similarities between MOGCTs and testicular germ cell tumors suggest that the strategies that are successful in treating testicular tumors may be applied to MOGCTs; furthermore, the vast majority of testicular tumors are germ cell tumors, which are much more common than MOGCTs [37,72]. Trials of unselected testicular tumor patients with advanced or platinum-resistant disease have not shown benefit of VEGF-tyrosine kinase inhibitors (TKI), EGFR-TKIs, or c-Kit inhibitors.

Imatinib, an inhibitor of several tyrosine kinases, including c-KIT, was evaluated in six patients with KIT-expressing refractory germ cell tumors [149]. A decline in AFP was seen in a single patient, who had stable disease for 3 months. A case report outlined a complete response in one heavily pretreated testicular tumor patient [150]. Pazopanib, a multikinase inhibitor with activity against VEGF receptors, c-KIT, and additional kinases, did not meet its primary endpoint of progression-free survival (PFS) in a single-arm trial including 43 patients who had failed at least two platinum-containing chemotherapy regimens [151]. Approximately 70% of patients had a short-lived decrease in tumor markers, but the overall response rate (ORR) was less than 5% [151].

Sunitinib also led to tumor marker decline, but showed no clinical benefit in a trial with ten patients [128]. However, in another trial, a single patient with chemotherapy-refractory testicular germ cell tumor had a clinical and biochemical response to sunitinib that lasted 17 months, potentially related to RET amplification in his tumor [152]. The combination of bevacizumab and oxaliplatin failed to meet the primary endpoint of PFS in a trial of 29 patients with chemotherapy-refractory testicular germ cell tumors [153]. Tivantinib, a different tyrosine receptor kinase inhibitor, failed to achieve any responses in patients with relapsed or refractory germ cell tumors [154].

The mammalian target of rapamycin (mTOR) inhibitor, everolimus, elicited no clinically meaningful responses as a single agent for patients with relapsed or platinum-refractory disease [155]. A trial of avelumab, a PD-L1 checkpoint inhibitor, in a similar setting also failed to meet its PFS endpoint [156], as did a trial of pembrolizumab [136]. Brentuximab vedotin, an antibody–drug conjugate targeting CD30, was evaluated in patients with chemotherapy-refractory tumors with an embryonal carcinoma component and expression of CD30 [157]. A complete and very good partial response was described.

## 6. Conclusions and Future Directions

Insights into the pathogenesis, molecular features, and omics of non-epithelial ovarian tumors have been accumulating in the recent years. The leading targeted therapy candidates from these translational and bench studies have been evaluated mainly in male germ cell tumors, without encouraging results. This exemplifies the fact that the vast majority of drug–indication pairs that are tested clinically do not achieve their expected clinical benefits and that many cancer therapies exert their effect in ways that are different from their presumed mechanisms [158]. A small number of clinical trials of females reflect paucity of potential indications for rare tumors that are often cured with surgery and chemotherapy. Recruitment of patients has depended on large referral centers or collaborative efforts. An additional challenge is to achieve diagnostic accuracy for atypical cases, which may require incorporation of mutational analysis, as demonstrated above for SCSTs.

Additional potential vulnerabilities of MOGCTs and SCSTs have been described, but have not been targeted in clinical trials. These include alterations that are common across multiple cancers, such as aneuploidy, *TERT* mutations, and activation of the RAS-RAF-MEK-ERK/JNK and PI3K/AKT/mTOR pathways [59,60]. Targeting these alterations does not rely solely on recruitment of non-epithelial ovarian tumor patients; trials of other solid tumors, including testicular tumors, may inform the treatment for MOGCTs and SCSTs. Other alterations are fairly specific to subtypes of non-epithelial ovarian tumors, including the recurrent *FOXL2* and common *DICER1* mutations in adult granulosa cell tumors and Sertoli–Leydig cell tumors, respectively [59,60]. Including the analysis of these and other mutations in prospective clinical trials may help delineate differences in the clinical course and response to therapy of specific molecular subtypes. Many features of non-epithelial ovarian tumors remain understudied, including hallmarks of chemotherapy-refractory disease [159], the interaction with the tumor microenvironment, the immune response, and more. The functional effects of tumor markers is also understudied, but have been shown to potentially promote tumor progression in other tumors [160].

Future studies should focus on effective and collaborative clinical trial designs that minimize the number of participants needed, e.g., multi-arm trials [161]. Clinical and molecular characterization of patients may lead to identification of prognostic and predictive biomarkers and may also detect rare somatic mutations that can be targeted through precision medicine initiatives and basket trials [162,163]. If effective therapies for chemotherapy-refractory disease are identified, they could potentially be leveraged to decrease the need for chemotherapy in the upfront setting and reduce long-term toxicity of common platinum-based regimens. 

## Figures and Tables

**Table 1:** Non-epithelial ovarian malignancies and their common genetic alterations.

Histological Subtypes	Common Genetic Alterations
*Malignant germ cell tumors*	*Low mutational burden, marked aneuploidy*
Dysgerminoma	*KIT* mutations (30–50%) Chromosome 12p gain (contains *KRAS*) (up to 80%)
Immature teratoma	Copy-neutral LOH * (100%)
Yolk sac tumor	*PI3K/AKT/mTOR* amplifications (~40%) *KIT* mutations (~55%) Chromosome 12p gain (contains *KRAS*) (~60%) TGF-β/BMP and Wnt/β-catenin signaling
Embryonal carcinoma	CD30 expression (~80%)
Mixed germ cell tumor	Chromosome 12p gain (contains *KRAS*) (~40%)
Choriocarcinoma	Wnt/β-catenin signaling
*Sex cord–stromal tumors*	
Adult granulosa cell tumor	*FOXL2* C134W mutation (> 95% of tumors) *TERT* mutations (~40%) *AKT1* amplification (~60%) Trisomy 8, 9, 12, or 14 Monosomy 22, 16
Juvenile granulosa cell tumor	*AKT1* duplication/activation (~60%) *GNAS* mutations (gsp) (~30%) Germline *IDH1/2* (Ollier disease, Maffucci syndrome) (rare)
Sertoli–Leydig cell tumor	Germline and somatic *DICER1* mutations (60%)
Sex cord–stromal tumors with annular tubules	Germline *STK11/LKB1* (Peutz–Jeghers syndrome) (~40%)
Sex cord–stromal tumors, NOS ^#^	No characteristic alterations described
Pure stromal or other pure sex cord tumors	

Note: The most common subtypes of malignant ovarian germ cell tumors and malignant ovarian sex cord–stromal tumors and their corresponding commonly identified alterations are noted [40,41,42]. Frequencies are estimates based on the available data, which are limited for certain alterations or tumor subtypes. * LOH—loss of heterozygosity. ^#^ NOS—not otherwise specified. Gsp—Gs-Protein, referring to the alpha subunit of G-protein (Gs).

**Table 2:** Clinical trials of targeted therapies for female MOGCTs and SCSTs.

Agent	Class	Indication	Female Patients (%)	Results	NCT
Imatinib	Kinase inhibitor	Relapsed/refractory stage II or stage III testicular or ovarian tumors	NA	NA	NCT00042952
Durvalumab/tremelimumab	Immunotherapy	Relapsed/refractory germ cell tumors	NA	NA	NCT03158064
Guadecitabine/cisplatin	Hypomethylating agent/chemotherapy	Relapsed/refractory germ cell tumors	1 (7%)	ORR 28%, 2/14 with CR	NCT02429466
Alvocidib/oxaliplatin± 5 FU	CDK9 inhibitor/chemotherapy	Relapsed/refractory germ cell tumors	1 (2.8%)	Primary endpoint not met	NCT00957905
Bevacizumab/Paclitaxel	Anti-angiogenesis/chemotherapy	Relapsed ovarian sex cord–stromal tumors	60 (100%)	No improvement in PFS	NCT01770301
Bevacizumab	Anti-angiogenesis	Relapsed ovarian sex cord–stromal tumors	36 (100%)	ORR 17%, SD 78%	NCT00748657
Ketoconazole	CYP17 inhibitor, antifungal agent	Locally advanced or metastatic granulosa cell tumor	6 (100%)	No responses, stable disease achieved in five patients	NCT01584297
Orteronel	CYP17 inhibitor, nonsteroidal drug	Locally advanced or metastatic granulosa cell tumor	10 (100%)	Three patients achieved stable disease for more than 12 months	NCT02101684
Onapristone	Progesterone antagonist	PR+, low-grade ovarian tumors, including granulosa cell tumors	84 (100%)	NA	NCT03909152
Enzalutamide	Androgen receptor signaling inhibitor	Locally advanced or metastatic granulosa cell tumor	35 (100%)	NA	NCT03464201
Anastrozole	Aromatase inhibitor	ER/PR+ recurrent/metastatic granulosa cell tumors of the ovary	41 (100%)	9.8% partial response, 59% progression-free at 6 months	ACTRN12610000796088

Clinical trials of targeted therapies for MOGCTs and SCSTs are summarized in the table. Trials without female participants are not listed. Abbreviations: ORR—overall response rate, CR—complete response, SD—stable disease, PFS—progression-free survival, PR—progesterone receptor. NA—not available. NCT—National Clinical Trial. 5FU—fluorouracil

## References

[1] Serov S.F., Scully R.E., Sobin L.J. (1973). Histological typing of ovarian tumors in World Health Organization. Proceedings of International Histological Classification of Tumors, Geneva, Switzerland.

[2] Kurman R., Carcangiu M., Herrington C., Young R. (2014). World Health Organization Classification of Tumours of Female Reproductive Organs.

[3] Torre L.A., Trabert B., DeSantis C.E., Miller K.D., Samimi G., Runowicz C.D., Gaudet M.M., Jemal A., Siegel R.L. (2018). Ovarian cancer statistics, 2018. CA Cancer J. Clin..

[4] Testa U., Petrucci E., Pasquini L., Castelli G., Pelosi E. (2018). Ovarian Cancers: Genetic Abnormalities, Tumor Heterogeneity and Progression, Clonal Evolution and Cancer Stem Cells. Medicines (Basel).

[5] Matz M., Coleman M.P., Sant M., Chirlaque M.D., Visser O., Gore M., Allemani C. (2017). The histology of ovarian cancer: Worldwide distribution and implications for international survival comparisons (CONCORD-2). Gynecol. Oncol..

[6] Schultz K.A., Harris A.K., Schneider D.T., Young R.H., Brown J., Gershenson D.M., Dehner L.P., Hill D.A., Messinger Y.H., Frazier A.L. (2016). Ovarian Sex Cord-Stromal Tumors. J. Oncol. Pract..

[7] Gershenson D.M. (2007). Management of ovarian germ cell tumors. J. Clin. Oncol..

[8] Colombo N., Parma G., Zanagnolo V., Insinga A. (2007). Management of ovarian stromal cell tumors. J. Clin. Oncol..

[9] Palenzuela G., Martin E., Meunier A., Beuzeboc P., Laurence V., Orbach D., Frappaz D. (2008). Comprehensive staging allows for excellent outcome in patients with localized malignant germ cell tumor of the ovary. Ann. Surg..

[10] Lee C.W., Song M.J., Park S.T., Ki E.Y., Lee S.J., Lee K.H., Ryu K.S., Park J.S., Hur S.Y. (2011). Residual tumor after the salvage surgery is the major risk factors for primary treatment failure in malignant ovarian germ cell tumors: A retrospective study of single institution. World J. Surg. Oncol..

[11] Mangili G., Sigismondi C., Lorusso D., Cormio G., Candiani M., Scarfone G., Mascilini F., Gadducci A., Mosconi A.M., Scollo P. (2017). The role of staging and adjuvant chemotherapy in stage I malignant ovarian germ cell tumors (MOGTs): The MITO-9 study. Ann. Oncol..

[12] Bristow R.E., Karlan B.Y., Chi D.S. (2015). Surgery for Ovarian Cancer.

[13] Ray-Coquard I., Morice P., Lorusso D., Prat J., Oaknin A., Pautier P., Colombo N. (2018). Non-epithelial ovarian cancer: ESMO Clinical Practice Guidelines for diagnosis, treatment and follow-up. Ann. Oncol..

[14] Billmire D., Vinocur C., Rescorla F., Cushing B., London W., Schlatter M., Davis M., Giller R., Lauer S., Olson T. (2004). Outcome and staging evaluation in malignant germ cell tumors of the ovary in children and adolescents: An intergroup study. J. Pediatr. Surg..

[15] Liu Q., Ding X., Yang J., Cao D., Shen K., Lang J., Zhang G., Xin X., Xie X., Wu Y. (2013). The significance of comprehensive staging surgery in malignant ovarian germ cell tumors. Gynecol. Oncol..

[16] Thomas G.M., Dembo A.J., Hacker N.F., DePetrillo A.D. (1987). Current therapy for dysgerminoma of the ovary. Obstet. Gynecol..

[17] Mangili G., Sigismondi C., Lorusso D., Cormio G., Scollo P., Vigano R., Gamucci T., Candiani M., Pignata S. (2011). Is surgical restaging indicated in apparent stage IA pure ovarian dysgerminoma? The MITO group retrospective experience. Gynecol. Oncol..

[18] Ceppi L., Galli F., Lamanna M., Magni S., Dell’Orto F., Verri D., Delle Marchette M., Lissoni A.A., Sina F., Giuliani D. (2019). Ovarian function, fertility, and menopause occurrence after fertility-sparing surgery and chemotherapy for ovarian neoplasms. Gynecol. Oncol..

[19] Sigismondi C., Scollo P., Ferrandina G., Candiani M., Angioli R., Vigano R., Scarfone G., Mangili G. (2015). Management of bilateral malignant ovarian germ cell tumors: A MITO-9 retrospective study. Int. J. Gynecol. Cancer.

[20] Yoo S.C., Kim W.Y., Yoon J.H., Chang S.J., Chang K.H., Ryu H.S. (2010). Young girls with malignant ovarian germ cell tumors can undergo normal menarche and menstruation after fertility-preserving surgery and adjuvant chemotherapy. Acta Obstet. Gynecol. Scand..

[21] Perrin L.C., Low J., Nicklin J.L., Ward B.G., Crandon A.J. (1999). Fertility and ovarian function after conservative surgery for germ cell tumours of the ovary. Aust. N. Z. J. Obstet. Gynaecol..

[22] Chan J.K., Tewari K.S., Waller S., Cheung M.K., Shin J.Y., Osann K., Kapp D.S. (2008). The influence of conservative surgical practices for malignant ovarian germ cell tumors. J. Surg. Oncol..

[23] Brown J., Sood A.K., Deavers M.T., Milojevic L., Gershenson D.M. (2009). Patterns of metastasis in sex cord-stromal tumors of the ovary: Can routine staging lymphadenectomy be omitted?. Gynecol. Oncol..

[24] Mahdi H., Swensen R.E., Hanna R., Kumar S., Ali-Fehmi R., Semaan A., Tamimi H., Morris R.T., Munkarah A.R. (2011). Prognostic impact of lymphadenectomy in clinically early stage malignant germ cell tumour of the ovary. Br. J. Cancer.

[25] Cheng H., Peng J., Yang Z., Zhang G. (2018). Prognostic significance of lymphadenectomyin malignant ovarian sex cord stromal tumor: A retrospective cohort study and meta-analysis. Gynecol. Oncol..

[26] Karalok A., Turan T., Ureyen I., Tasci T., Basaran D., Koc S., Boran N., Kose M.F., Tulunay G. (2016). Prognostic Factors in Adult Granulosa Cell Tumor: A Long Follow-Up at a Single Center. Int. J. Gynecol. Cancer.

[27] Kuru O., Boyraz G., Uckan H., Erturk A., Gultekin M., Ozgul N., Salman C., Yuce K. (2017). Retroperitoneal nodal metastasis in primary adult type granulosa cell tumor of the ovary: Can routine lymphadenectomy be omitted?. Eur. J. Obstet. Gynecol. Reprod. Biol..

[28] Erkilinc S., Taylan E., Karatasli V., Uzaldi I., Karadeniz T., Gokcu M., Sanci M. (2019). Does lymphadenectomy effect postoperative surgical morbidity and survival in patients with adult granulosa cell tumor of ovary?. J. Obstet. Gynaecol. Res..

[29] Colombo N., Peiretti M., Garbi A., Carinelli S., Marini C., Sessa C. (2012). Non-epithelial ovarian cancer: ESMO Clinical Practice Guidelines for diagnosis, treatment and follow-up. Ann. Oncol..

[30] De Giorgi U., Casadei C., Bergamini A., Attademo L., Cormio G., Lorusso D., Pignata S., Mangili G. (2019). Therapeutic Challenges for Cisplatin-Resistant Ovarian Germ Cell Tumors. Cancers (Basel).

[31] Leary A.F., Quinn M., Fujiwara K., Coleman R.L., Kohn E., Sugiyama T., Glasspool R., Ray-Coquard I., Colombo N., Bacon M. (2017). Fifth Ovarian Cancer Consensus Conference of the Gynecologic Cancer InterGroup (GCIG): Clinical trial design for rare ovarian tumours. Ann. Oncol..

[32] De Giorgi U., Richard S., Badoglio M., Kanfer E., Bourrhis J.H., Nicolas-Virelizier E., Vettenranta K., Lioure B., Martin S., Dreger P. (2017). Salvage high-dose chemotherapy in female patients with relapsed/refractory germ-cell tumors: A retrospective analysis of the European Group for Blood and Marrow Transplantation (EBMT). Ann. Oncol..

[33] Reddy Ammakkanavar N., Matei D., Abonour R., Einhorn L.H. (2015). High-dose chemotherapy for recurrent ovarian germ cell tumors. J. Clin. Oncol..

[34] Hubbard A.K., Poynter J.N. (2019). Global incidence comparisons and trends in ovarian germ cell tumors by geographic region in girls, adolescents and young women: 1988–2012. Gynecol. Oncol..

[35] Bryant C.S., Kumar S., Shah J.P., Mahdi H., Ali-Fehmi R., Munkarah A.R., Deppe G., Morris R.T. (2009). Racial disparities in survival among patients with germ cell tumors of the ovary–United States. Gynecol. Oncol..

[36] Smith H.O., Berwick M., Verschraegen C.F., Wiggins C., Lansing L., Muller C.Y., Qualls C.R. (2006). Incidence and survival rates for female malignant germ cell tumors. Obstet. Gynecol..

[37] Kraggerud S.M., Hoei-Hansen C.E., Alagaratnam S., Skotheim R.I., Abeler V.M., Rajpert-De Meyts E., Lothe R.A. (2013). Molecular characteristics of malignant ovarian germ cell tumors and comparison with testicular counterparts: Implications for pathogenesis. Endocr. Rev..

[38] Solheim O., Kaern J., Trope C.G., Rokkones E., Dahl A.A., Nesland J.M., Fossa S.D. (2013). Malignant ovarian germ cell tumors: Presentation, survival and second cancer in a population based Norwegian cohort (1953–2009). Gynecol. Oncol..

[39] Cicin I., Eralp Y., Saip P., Ayan I., Kebudi R., Iyibozkurt C., Tuzlali S., Gorgun O., Topuz E. (2009). Malignant ovarian germ cell tumors: A single-institution experience. Am. J. Clin. Oncol..

[40] Fuller P.J., Leung D., Chu S. (2017). Genetics and genomics of ovarian sex cord-stromal tumors. Clin. Genet..

[41] Van Nieuwenhuysen E., Lambrechts S., Lambrechts D., Leunen K., Amant F., Vergote I. (2013). Genetic changes in nonepithelial ovarian cancer. Expert Rev. Anticancer Ther..

[42] Van Nieuwenhuysen E., Busschaert P., Neven P., Han S.N., Moerman P., Liontos M., Papaspirou M., Kupryjanczyk J., Hogdall C., Hogdall E. (2018). The genetic landscape of 87 ovarian germ cell tumors. Gynecol. Oncol..

[43] Peccatori F., Bonazzi C., Chiari S., Landoni F., Colombo N., Mangioni C. (1995). Surgical management of malignant ovarian germ-cell tumors: 10 years’ experience of 129 patients. Obstet. Gynecol..

[44] Kurman R.J., Norris H.J. (1977). Malignant germ cell tumors of the ovary. Hum. Pathol..

[45] Barakat R.R.B., Berchuck A., Markman M., Randall M.E. (2013). Principles and Practice of Gynecologic Oncology.

[46] Newton C., Murali K., Ahmad A., Hockings H., Graham R., Liberale V., Sarker S.J., Ledermann J., Berney D.M., Shamash J. (2019). A multicentre retrospective cohort study of ovarian germ cell tumours: Evidence for chemotherapy de-escalation and alignment of paediatric and adult practice. Eur. J. Cancer.

[47] Williams S., Blessing J.A., Liao S.Y., Ball H., Hanjani P. (1994). Adjuvant therapy of ovarian germ cell tumors with cisplatin, etoposide, and bleomycin: A trial of the Gynecologic Oncology Group. J. Clin. Oncol..

[48] Gershenson D.M., Morris M., Cangir A., Kavanagh J.J., Stringer C.A., Edwards C.L., Silva E.G., Wharton J.T. (1990). Treatment of malignant germ cell tumors of the ovary with bleomycin, etoposide, and cisplatin. J. Clin. Oncol..

[49] Chovanec M., Abu Zaid M., Hanna N., El-Kouri N., Einhorn L.H., Albany C. (2017). Long-term toxicity of cisplatin in germ-cell tumor survivors. Ann. Oncol..

[50] Duhil de Benaze G., Pacquement H., Faure-Conter C., Patte C., Orbach D., Corradini N., Berger C., Sudour-Bonnange H., Verite C., Martelli H. (2018). Paediatric dysgerminoma: Results of three consecutive French germ cell tumours clinical studies (TGM-85/90/95) with late effects study. Eur. J. Cancer.

[51] De La Motte Rouge T., Pautier P., Duvillard P., Rey A., Morice P., Haie-Meder C., Kerbrat P., Culine S., Troalen F., Lhomme C. (2008). Survival and reproductive function of 52 women treated with surgery and bleomycin, etoposide, cisplatin (BEP) chemotherapy for ovarian yolk sac tumor. Ann. Oncol..

[52] Gershenson D.M., Miller A.M., Champion V.L., Monahan P.O., Zhao Q., Cella D., Williams S.D. (2007). Reproductive and sexual function after platinum-based chemotherapy in long-term ovarian germ cell tumor survivors: A Gynecologic Oncology Group Study. J. Clin. Oncol..

[53] Williams S.D., Kauderer J., Burnett A.F., Lentz S.S., Aghajanian C., Armstrong D.K. (2004). Adjuvant therapy of completely resected dysgerminoma with carboplatin and etoposide: A trial of the Gynecologic Oncology Group. Gynecol. Oncol..

[54] Bokemeyer C., Kohrmann O., Tischler J., Weissbach L., Rath U., Haupt A., Schoffski P., Harstrick A., Schmoll H.J. (1996). A randomized trial of cisplatin, etoposide and bleomycin (PEB) versus carboplatin, etoposide and bleomycin (CEB) for patients with ‘good-risk’ metastatic non-seminomatous germ cell tumors. Ann. Oncol..

[55] Pashankar F., Hale J.P., Dang H., Krailo M., Brady W.E., Rodriguez-Galindo C., Nicholson J.C., Murray M.J., Bilmire D.F., Stoneham S. (2016). Is adjuvant chemotherapy indicated in ovarian immature teratomas? A combined data analysis from the Malignant Germ Cell Tumor International Collaborative. Cancer.

[56] Heskett M.B., Sanborn J.Z., Boniface C., Goode B., Chapman J., Garg K., Rabban J.T., Zaloudek C., Benz S.C., Spellman P.T. (2020). Multiregion exome sequencing of ovarian immature teratomas reveals 2N near-diploid genomes, paucity of somatic mutations, and extensive allelic imbalances shared across mature, immature, and disseminated components. Mod. Pathol..

[57] Kroeger P.T., Drapkin R. (2017). Pathogenesis and heterogeneity of ovarian cancer. Curr. Opin. Obstet. Gynecol..

[58] Shen H., Shih J., Hollern D.P., Wang L., Bowlby R., Tickoo S.K., Thorsson V., Mungall A.J., Newton Y., Hegde A.M. (2018). Integrated Molecular Characterization of Testicular Germ Cell Tumors. Cell Rep..

[59] AACR Project GENIE Consortium (2017). AACR Project GENIE: Powering Precision Medicine through an International Consortium. Cancer Discov..

[60] Gao J., Aksoy B.A., Dogrusoz U., Dresdner G., Gross B., Sumer S.O., Sun Y., Jacobsen A., Sinha R., Larsson E. (2013). Integrative analysis of complex cancer genomics and clinical profiles using the cBioPortal. Sci. Signal..

[61] Zhao X., Wei Y.Q., Kariya Y., Teshigawara K., Uchida A. (1995). Accumulation of gamma/delta T cells in human dysgerminoma and seminoma: Roles in autologous tumor killing and granuloma formation. Immunol. Investig..

[62] Dietl J., Horny H.P., Ruck P., Kaiserling E. (1993). Dysgerminoma of the ovary. An immunohistochemical study of tumor-infiltrating lymphoreticular cells and tumor cells. Cancer.

[63] Stewart C.J., Farquharson M.A., Foulis A.K. (1992). Characterization of the inflammatory infiltrate in ovarian dysgerminoma: An immunocytochemical study. Histopathology.

[64] Honma S., Uchiyama M., Omomo Y., Watanabe S., Sasagawa M., Kanazawa K., Takeuchi S. (1986). Immunohistologic characterization of lymphoid cells infiltrating dysgerminoma of the ovary. Nihon Sanka Fujinka Gakkai Zasshi.

[65] Steffen R., Genton C.Y. (1980). The significance of lymphocytic infiltration for the prognosis in dysgerminomas. Schweiz. Med. Wochenschr..

[66] Willis S.N., Mallozzi S.S., Rodig S.J., Cronk K.M., McArdel S.L., Caron T., Pinkus G.S., Lovato L., Shampain K.L., Anderson D.E. (2009). The microenvironment of germ cell tumors harbors a prominent antigen-driven humoral response. J. Immunol..

[67] Germain C., Gnjatic S., Dieu-Nosjean M.C. (2015). Tertiary Lymphoid Structure-Associated B Cells are Key Players in Anti-Tumor Immunity. Front. Immunol..

[68] Dieu-Nosjean M.C., Goc J., Giraldo N.A., Sautes-Fridman C., Fridman W.H. (2014). Tertiary lymphoid structures in cancer and beyond. Trends Immunol..

[69] Maoz A., Dennis M., Greenson J.K. (2019). The Crohn’s-Like Lymphoid Reaction to Colorectal Cancer-Tertiary Lymphoid Structures with Immunologic and Potentially Therapeutic Relevance in Colorectal Cancer. Front. Immunol..

[70] Siska P.J., Johnpulle R.A.N., Zhou A., Bordeaux J., Kim J.Y., Dabbas B., Dakappagari N., Rathmell J.C., Rathmell W.K., Morgans A.K. (2017). Deep exploration of the immune infiltrate and outcome prediction in testicular cancer by quantitative multiplexed immunohistochemistry and gene expression profiling. Oncoimmunology.

[71] Fankhauser C.D., Curioni-Fontecedro A., Allmann V., Beyer J., Tischler V., Sulser T., Moch H., Bode P.K. (2015). Frequent PD-L1 expression in testicular germ cell tumors. Br. J. Cancer.

[72] Berney D.M., Stoneham S., Arora R., Shamash J., Lockley M. (2020). Ovarian germ cell tumour classification: Views from the testis. Histopathology.

[73] Lin K.Y., Bryant S., Miller D.S., Kehoe S.M., Richardson D.L., Lea J.S. (2014). Malignant ovarian germ cell tumor—Role of surgical staging and gonadal dysgenesis. Gynecol. Oncol..

[74] Scully R.E. (1970). Gonadoblastoma. A review of 74 cases. Cancer.

[75] Pleskacova J., Hersmus R., Oosterhuis J.W., Setyawati B.A., Faradz S.M., Cools M., Wolffenbuttel K.P., Lebl J., Drop S.L., Looijenga L.H. (2010). Tumor risk in disorders of sex development. Sex. Dev..

[76] Cheng L., Roth L.M., Zhang S., Wang M., Morton M.J., Zheng W., Abdul Karim F.W., Montironi R., Lopez-Beltran A. (2011). KIT gene mutation and amplification in dysgerminoma of the ovary. Cancer.

[77] Cardoso H.J., Figueira M.I., Socorro S. (2017). The stem cell factor (SCF)/c-KIT signalling in testis and prostate cancer. J. Cell Commun. Signal..

[78] Heinrich M.C., Corless C.L., Demetri G.D., Blanke C.D., von Mehren M., Joensuu H., McGreevey L.S., Chen C.J., Van den Abbeele A.D., Druker B.J. (2003). Kinase mutations and imatinib response in patients with metastatic gastrointestinal stromal tumor. J. Clin. Oncol..

[79] Cossu-Rocca P., Zhang S., Roth L.M., Eble J.N., Zheng W., Karim F.W., Michael H., Emerson R.E., Jones T.D., Hattab E.M. (2006). Chromosome 12p abnormalities in dysgerminoma of the ovary: A FISH analysis. Mod. Pathol..

[80] McCormick F. (2015). KRAS as a Therapeutic Target. Clin. Cancer Res..

[81] Maoz A., Ciccone M.A., Matsuzaki S., Coleman R.L., Matsuo K. (2019). Emerging serine-threonine kinase inhibitors for treating ovarian cancer. Expert Opin. Emerg. Drugs.

[82] Gershenson D.M. (2012). Current advances in the management of malignant germ cell and sex cord-stromal tumors of the ovary. Gynecol. Oncol..

[83] Gershenson D.M., Okamoto A., Ray-Coquard I. (2019). Management of Rare Ovarian Cancer Histologies. J. Clin. Oncol..

[84] Ciccone M.A., Maoz A., Casabar J.K., Machida H., Mabuchi S., Matsuo K. (2016). Clinical outcome of treatment with serine-threonine kinase inhibitors in recurrent epithelial ovarian cancer: A systematic review of literature. Expert Opin. Investig. Drugs.

[85] Mabuchi S., Kuroda H., Takahashi R., Sasano T. (2015). The PI3K/AKT/mTOR pathway as a therapeutic target in ovarian cancer. Gynecol. Oncol..

[86] Krishnamurthy N., Kurzrock R. (2018). Targeting the Wnt/beta-catenin pathway in cancer: Update on effectors and inhibitors. Cancer Treat. Rev..

[87] Quirk J.T., Natarajan N. (2005). Ovarian cancer incidence in the United States, 1992–1999. Gynecol. Oncol..

[88] Rabban J.T., Zaloudek C.J. (2013). A practical approach to immunohistochemical diagnosis of ovarian germ cell tumours and sex cord-stromal tumours. Histopathology.

[89] Sung M.T., Jones T.D., Beck S.D., Foster R.S., Cheng L. (2006). OCT4 is superior to CD30 in the diagnosis of metastatic embryonal carcinomas after chemotherapy. Hum. Pathol..

[90] Leroy X., Augusto D., Leteurtre E., Gosselin B. (2002). CD30 and CD117 (c-kit) used in combination are useful for distinguishing embryonal carcinoma from seminoma. J. Histochem. Cytochem..

[91] Brown J., Shvartsman H.S., Deavers M.T., Burke T.W., Munsell M.F., Gershenson D.M. (2004). The Activity of Taxanes in the Treatment of Sex Cord-Stromal Ovarian Tumors. J. Clin. Oncol..

[92] Thrall M.M., Paley P., Pizer E., Garcia R., Goff B.A. (2011). Patterns of spread and recurrence of sex cord-stromal tumors of the ovary. Gynecol. Oncol..

[93] Zhang M., Cheung M.K., Shin J.Y., Kapp D.S., Husain A., Teng N.N., Berek J.S., Osann K., Chan J.K. (2007). Prognostic factors responsible for survival in sex cord stromal tumors of the ovary–An analysis of 376 women. Gynecol. Oncol..

[94] Shah S.P., Kobel M., Senz J., Morin R.D., Clarke B.A., Wiegand K.C., Leung G., Zayed A., Mehl E., Kalloger S.E. (2009). Mutation of FOXL2 in granulosa-cell tumors of the ovary. N. Engl. J. Med..

[95] Kommoss F., Lehr H.A. (2019). Sex cord-stromal tumors of the ovary: Current aspects with a focus on granulosa cell tumors, Sertoli-Leydig cell tumors, and gynandroblastomas. Pathologe.

[96] Fox H., Agrawal K., Langley F.A. (1975). A clinicopathologic study of 92 cases of granulosa cell tumor of the ovary with special reference to the factors influencing prognosis. Cancer.

[97] Ohel G., Kaneti H., Schenker J.G. (1983). Granulosa cell tumors in Israel: A study of 172 cases. Gynecol. Oncol..

[98] Yanagida S., Anglesio M.S., Nazeran T.M., Lum A., Inoue M., Iida Y., Takano H., Nikaido T., Okamoto A., Huntsman D.G. (2017). Clinical and genetic analysis of recurrent adult-type granulosa cell tumor of the ovary: Persistent preservation of heterozygous c.402C > G FOXL2 mutation. PLoS ONE.

[99] Karnezis A.N., Wang Y., Keul J., Tessier-Cloutier B., Magrill J., Kommoss S., Senz J., Yang W., Proctor L., Schmidt D. (2019). DICER1 and FOXL2 Mutation Status Correlates with Clinicopathologic Features in Ovarian Sertoli-Leydig Cell Tumors. Am. J. Surg. Pathol..

[100] Kommoss S., Anglesio M.S., Mackenzie R., Yang W., Senz J., Ho J., Bell L., Lee S., Lorette J., Huntsman D.G. (2013). FOXL2 molecular testing in ovarian neoplasms: Diagnostic approach and procedural guidelines. Mod. Pathol..

[101] Jamieson S., Butzow R., Andersson N., Alexiadis M., Unkila-Kallio L., Heikinheimo M., Fuller P.J., Anttonen M. (2010). The FOXL2 C134W mutation is characteristic of adult granulosa cell tumors of the ovary. Mod. Pathol..

[102] Leung D.T.H., Fuller P.J., Chu S. (2016). Impact of FOXL2 mutations on signaling in ovarian granulosa cell tumors. Int. J. Biochem. Cell Biol..

[103] Georges A., L’Hote D., Todeschini A.L., Auguste A., Legois B., Zider A., Veitia R.A. (2014). The transcription factor FOXL2 mobilizes estrogen signaling to maintain the identity of ovarian granulosa cells. eLife.

[104] Benayoun B.A., Anttonen M., L’Hote D., Bailly-Bechet M., Andersson N., Heikinheimo M., Veitia R.A. (2013). Adult ovarian granulosa cell tumor transcriptomics: Prevalence of FOXL2 target genes misregulation gives insights into the pathogenic mechanism of the p.Cys134Trp somatic mutation. Oncogene.

[105] Rabban J.T., Karnezis A.N., Devine W.P. (2020). Practical roles for molecular diagnostic testing in ovarian adult granulosa cell tumour, Sertoli-Leydig cell tumour, microcystic stromal tumour and their mimics. Histopathology.

[106] Park M., Shin E., Won M., Kim J.H., Go H., Kim H.L., Ko J.J., Lee K., Bae J. (2010). FOXL2 interacts with steroidogenic factor-1 (SF-1) and represses SF-1-induced CYP17 transcription in granulosa cells. Mol. Endocrinol..

[107] Fleming N.I., Knower K.C., Lazarus K.A., Fuller P.J., Simpson E.R., Clyne C.D. (2010). Aromatase is a direct target of FOXL2: C134W in granulosa cell tumors via a single highly conserved binding site in the ovarian specific promoter. PLoS ONE.

[108] Alexiadis M., Rowley S.M., Chu S., Leung D.T.H., Stewart C.J.R., Amarasinghe K.C., Campbell I.G., Fuller P.J. (2019). Mutational Landscape of Ovarian Adult Granulosa Cell Tumors from Whole Exome and Targeted TERT Promoter Sequencing. Mol. Cancer Res..

[109] Pilsworth J.A., Cochrane D.R., Xia Z., Aubert G., Farkkila A.E.M., Horlings H.M., Yanagida S., Yang W., Lim J.L.P., Wang Y.K. (2018). TERT promoter mutation in adult granulosa cell tumor of the ovary. Mod. Pathol..

[110] Wang Y.K., Bashashati A., Anglesio M.S., Cochrane D.R., Grewal D.S., Ha G., McPherson A., Horlings H.M., Senz J., Prentice L.M. (2017). Genomic consequences of aberrant DNA repair mechanisms stratify ovarian cancer histotypes. Nat. Genet..

[111] Mayr D., Kaltz-Wittmer C., Arbogast S., Amann G., Aust D.E., Diebold J. (2002). Characteristic pattern of genetic aberrations in ovarian granulosa cell tumors. Mod. Pathol..

[112] Caburet S., Anttonen M., Todeschini A.L., Unkila-Kallio L., Mestivier D., Butzow R., Veitia R.A. (2015). Combined comparative genomic hybridization and transcriptomic analyses of ovarian granulosa cell tumors point to novel candidate driver genes. BMC Cancer.

[113] Kalfa N., Ecochard A., Patte C., Duvillard P., Audran F., Pienkowski C., Thibaud E., Brauner R., Lecointre C., Plantaz D. (2006). Activating mutations of the stimulatory g protein in juvenile ovarian granulosa cell tumors: A new prognostic factor?. J. Clin. Endocrinol. Metab..

[114] Bessiere L., Todeschini A.L., Auguste A., Sarnacki S., Flatters D., Legois B., Sultan C., Kalfa N., Galmiche L., Veitia R.A. (2015). A Hot-spot of In-frame Duplications Activates the Oncoprotein AKT1 in Juvenile Granulosa Cell Tumors. EBioMedicine.

[115] Hunzicker-Dunn M., Maizels E.T. (2006). FSH signaling pathways in immature granulosa cells that regulate target gene expression: Branching out from protein kinase A. Cell Signal..

[116] Farkkila A., Anttonen M., Pociuviene J., Leminen A., Butzow R., Heikinheimo M., Unkila-Kallio L. (2011). Vascular endothelial growth factor (VEGF) and its receptor VEGFR-2 are highly expressed in ovarian granulosa cell tumors. Eur. J. Endocrinol..

[117] Farkkila A., Pihlajoki M., Tauriala H., Butzow R., Leminen A., Unkila-Kallio L., Heikinheimo M., Anttonen M. (2011). Serum vascular endothelial growth factor A (VEGF) is elevated in patients with ovarian granulosa cell tumor (GCT), and VEGF inhibition by bevacizumab induces apoptosis in GCT in vitro. J. Clin. Endocrinol. Metab..

[118] Schmidt M., Kammerer U., Segerer S., Cramer A., Kohrenhagen N., Dietl J., Voelker H.U. (2008). Glucose metabolism and angiogenesis in granulosa cell tumors of the ovary: Activation of Akt, expression of M2PK, TKTL1 and VEGF. Eur. J. Obstet. Gynecol. Reprod. Biol..

[119] Rocconi R.P., Matthews K.S., Kimball K.J., Conner M.G., Baker A.C., Barnes M.N. (2008). Expression of c-kit and platelet-derived growth factor receptors in ovarian granulosa cell tumors. Reprod. Sci..

[120] Heravi-Moussavi A., Anglesio M.S., Cheng S.W., Senz J., Yang W., Prentice L., Fejes A.P., Chow C., Tone A., Kalloger S.E. (2012). Recurrent somatic DICER1 mutations in nonepithelial ovarian cancers. N. Engl. J. Med..

[121] de Kock L., Terzic T., McCluggage W.G., Stewart C.J.R., Shaw P., Foulkes W.D., Clarke B.A. (2017). DICER1 Mutations Are Consistently Present in Moderately and Poorly Differentiated Sertoli-Leydig Cell Tumors. Am. J. Surg. Pathol..

[122] Schultz K.A., Pacheco M.C., Yang J., Williams G.M., Messinger Y., Hill D.A., Dehner L.P., Priest J.R. (2011). Ovarian sex cord-stromal tumors, pleuropulmonary blastoma and DICER1 mutations: A report from the International Pleuropulmonary Blastoma Registry. Gynecol. Oncol..

[123] Goulvent T., Ray-Coquard I., Borel S., Haddad V., Devouassoux-Shisheboran M., Vacher-Lavenu M.C., Pujade-Laurraine E., Savina A., Maillet D., Gillet G. (2016). DICER1 and FOXL2 mutations in ovarian sex cord-stromal tumours: A GINECO Group study. Histopathology.

[124] Young R.H., Welch W.R., Dickersin G.R., Scully R.E. (1982). Ovarian sex cord tumor with annular tubules: Review of 74 cases including 27 with Peutz-Jeghers syndrome and four with adenoma malignum of the cervix. Cancer.

[125] Fleuren E.D., Zhang L., Wu J., Daly R.J. (2016). The kinome ‘at large’ in cancer. Nat. Rev. Cancer.

[126] Raspagliesi F., Martinelli F., Grijuela B., Guadalupi V. (2011). Third-line chemotherapy with tyrosine kinase inhibitor (imatinib mesylate) in recurrent ovarian granulosa cell tumor: Case report. J. Obstet. Gynaecol. Res..

[127] Poddubskaya E.V., Baranova M.P., Allina D.O., Sekacheva M.I., Makovskaia L.A., Kamashev D.E., Suntsova M.V., Barbara V.S., Kochergina-Nikitskaya I.N., Aleshin A.A. (2019). Personalized prescription of imatinib in recurrent granulosa cell tumor of the ovary: Case report. Cold Spring Harb. Mol. Case Stud..

[128] Feldman D.R., Turkula S., Ginsberg M.S., Ishill N., Patil S., Carousso M., Bosl G.J., Motzer R.J. (2010). Phase II trial of sunitinib in patients with relapsed or refractory germ cell tumors. Investig. New Drugs.

[129] Manchana T., Ittiwut C., Mutirangura A., Kavanagh J.J. (2010). Targeted therapies for rare gynaecological cancers. Lancet Oncol..

[130] Brown J., Brady W.E., Schink J., Van Le L., Leitao M., Yamada S.D., de Geest K., Gershenson D.M. (2014). Efficacy and safety of bevacizumab in recurrent sex cord-stromal ovarian tumors: Results of a phase 2 trial of the Gynecologic Oncology Group. Cancer.

[131] Tao X., Sood A.K., Deavers M.T., Schmeler K.M., Nick A.M., Coleman R.L., Milojevic L., Gershenson D.M., Brown J. (2009). Anti-angiogenesis therapy with bevacizumab for patients with ovarian granulosa cell tumors. Gynecol. Oncol..

[132] Ray-Coquard I.L., Harter P., Lorusso D., Dalban C., Vergote I.B., Fujiwara K., Gladieff L., Lueck H.J., Floquet A., Lesoin A.F. (2018). Alienor/ENGOT-ov7 randomized trial exploring weekly paclitaxel (wP) + bevacizumab (bev) vs. wP alone for patients with ovarian sex cord tumors (SCT) in relapse. Ann. Oncol..

[133] Duffy M.J., Crown J. (2019). Biomarkers for Predicting Response to Immunotherapy with Immune Checkpoint Inhibitors in Cancer Patients. Clin. Chem..

[134] Thorsson V., Gibbs D.L., Brown S.D., Wolf D., Bortone D.S., Ou Yang T.H., Porta-Pardo E., Gao G.F., Plaisier C.L., Eddy J.A. (2018). The Immune Landscape of Cancer. Immunity.

[135] Herzog T., Arguello D., Reddy S., Gatalica Z. (2015). PD-1 and PD-L1 expression in 1599 gynecological malignancies-implications for immunotherapy. Gynecol. Oncol..

[136] Adra N., Einhorn L.H., Althouse S.K., Ammakkanavar N.R., Musapatika D., Albany C., Vaughn D., Hanna N.H. (2018). Phase II trial of pembrolizumab in patients with platinum refractory germ-cell tumors: A Hoosier Cancer Research Network Study GU14-206. Ann. Oncol..

[137] Rosenberg S.A., Tran E., Robbins P.F. (2017). T-Cell Transfer Therapy Targeting Mutant KRAS. N. Engl. J. Med..

[138] Hong L.K., Chen Y., Smith C.C., Montgomery S.A., Vincent B.G., Dotti G., Savoldo B. (2018). CD30-Redirected Chimeric Antigen Receptor T Cells Target CD30(+) and CD30(-) Embryonal Carcinoma via Antigen-Dependent and Fas/FasL Interactions. Cancer Immunol. Res..

[139] van Meurs H.S., van der Velden J., Buist M.R., van Driel W.J., Kenter G.G., van Lonkhuijzen L.R. (2015). Evaluation of response to hormone therapy in patients with measurable adult granulosa cell tumors of the ovary. Acta Obstet. Gynecol. Scand..

[140] Fishman A., Kudelka A.P., Tresukosol D., Edwards C.L., Freedman R.S., Kaplan A.L., Girtanner R.E., Kavanagh J.J. (1996). Leuprolide acetate for treating refractory or persistent ovarian granulosa cell tumor. J. Reprod. Med..

[141] Freeman S.A., Modesitt S.C. (2006). Anastrozole therapy in recurrent ovarian adult granulosa cell tumors: A report of 2 cases. Gynecol. Oncol..

[142] van Meurs H.S., van Lonkhuijzen L.R.C.W., Limpens J., van der Velden J., Buist M.R. (2014). Hormone therapy in ovarian granulosa cell tumors: A systematic review. Gynecol. Oncol..

[143] Yang A.D., Curtin J., Muggia F. (2018). Ovarian adult-type granulosa cell tumor: Focusing on endocrine-based therapies. Int. J. Endocr. Oncol..

[144] Banerjee S.N., Tang M., O’Connell R., Clamp A.R., Lord R., Mullassery V.M., Hall M., Gourley C., Bonaventura T., Goh J.C. (2018). PARAGON: A phase 2 study of anastrozole (An) in patients with estrogen receptor(ER) and/progesterone receptor (PR) positive recurrent/metastatic granulosa cell tumors/sex-cord stromal tumors (GCT) of the ovary. J. Clin. Oncol..

[145] Garcia-Donas J., Hurtado A., Garcia-Casado Z., Albareda J., Lopez-Guerrero J.A., Alemany I., Grande E., Camara J.C., Hernando S. (2013). Cytochrome P17 inhibition with ketoconazole as treatment for advanced granulosa cell ovarian tumor. J. Clin. Oncol..

[146] Garcia-Donas J., Hurtado A., Garrigos L., Santaballa A., Redondo A., Vidal L., Lainez N., Guerra E., Rodriguez V., Cueva J. Open Label Phase III Clinical Trial of Ketoconazole as CYP17 Inhibitor in Metastatic or Advanced Non-Resectable Granulosa Cell Ovarian Tumors. The GREKO (GRanulosa Et KetOconazole) Trial. GETHI 2011-03. https://ssrn.com/abstract=3377550.

[147] García-Donas J., Garrigos L., Lainez N., Santaballa A., Redondo A., Cueva J.F., Rubio M.J., Prieto M., Lopez-Guerrero J.A., Garcia-Casado Z. (2017). Open label phase II clinical trial of orteronel (TAK-700) in metastatic or advanced non-resectable granulosa cell ovarian tumors: The Greko II study. J. Clin. Oncol..

[148] Kawakami M., Liu X., Dmitrovsky E. (2019). New Cell Cycle Inhibitors Target Aneuploidy in Cancer Therapy. Annu. Rev. Pharmacol. Toxicol..

[149] Einhorn L.H., Brames M.J., Heinrich M.C., Corless C.L., Madani A. (2006). Phase II study of imatinib mesylate in chemotherapy refractory germ cell tumors expressing KIT. Am. J. Clin. Oncol..

[150] Pedersini R., Vattemi E., Mazzoleni G., Graiff C. (2007). Complete response after treatment with imatinib in pretreated disseminated testicular seminoma with overexpression of c-KIT. Lancet Oncol..

[151] Necchi A., Lo Vullo S., Giannatempo P., Raggi D., Calareso G., Togliardi E., Crippa F., Pennati M., Zaffaroni N., Perrone F. (2017). Pazopanib in advanced germ cell tumors after chemotherapy failure: Results of the open-label, single-arm, phase 2 Pazotest trial. Ann. Oncol..

[152] Subbiah V., Meric-Bernstam F., Mills G.B., Shaw K.R., Bailey A.M., Rao P., Ward J.F., Pagliaro L.C. (2014). Next generation sequencing analysis of platinum refractory advanced germ cell tumor sensitive to Sunitinib (Sutent(R)) a VEGFR2/PDGFRbeta/c-kit/ FLT3/RET/CSF1R inhibitor in a phase II trial. J. Hematol. Oncol..

[153] Jain A., Brames M.J., Vaughn D.J., Einhorn L.H. (2014). Phase II clinical trial of oxaliplatin and bevacizumab in refractory germ cell tumors. Am. J. Clin. Oncol..

[154] Feldman D.R., Einhorn L.H., Quinn D.I., Loriot Y., Joffe J.K., Vaughn D.J., Flechon A., Hajdenberg J., Halim A.B., Zahir H. (2013). A phase 2 multicenter study of tivantinib (ARQ 197) monotherapy in patients with relapsed or refractory germ cell tumors. Investig. New Drugs.

[155] Fenner M., Oing C., Dieing A., Gauler T., Oechsle K., Lorch A., Hentrich M., Kopp H.G., Bokemeyer C., Honecker F. (2019). Everolimus in patients with multiply relapsed or cisplatin refractory germ cell tumors: Results of a phase II, single-arm, open-label multicenter trial (RADIT) of the German Testicular Cancer Study Group. J. Cancer Res. Clin. Oncol..

[156] Mego M., Svetlovska D., Chovanec M., Reckova M., Rejlekova K., Obertova J., Palacka P., Sycova-Mila Z., De Giorgi U., Mardiak J. (2019). Phase II study of avelumab in multiple relapsed/refractory germ cell cancer. Investig. New Drugs.

[157] Albany C., Einhorn L., Garbo L., Boyd T., Josephson N., Feldman D.R. (2018). Treatment of CD30-Expressing Germ Cell Tumors and Sex Cord Stromal Tumors with Brentuximab Vedotin: Identification and Report of Seven Cases. Oncologist.

[158] Lin A., Giuliano C.J., Palladino A., John K.M., Abramowicz C., Yuan M.L., Sausville E.L., Lukow D.A., Liu L., Chait A.R. (2019). Off-target toxicity is a common mechanism of action of cancer drugs undergoing clinical trials. Sci. Transl. Med..

[159] Taylor-Weiner A., Zack T., O’Donnell E., Guerriero J.L., Bernard B., Reddy A., Han G.C., AlDubayan S., Amin-Mansour A., Schumacher S.E. (2016). Genomic evolution and chemoresistance in germ-cell tumours. Nature.

[160] Engle D.D., Tiriac H., Rivera K.D., Pommier A., Whalen S., Oni T.E., Alagesan B., Lee E.J., Yao M.A., Lucito M.S. (2019). The glycan CA19-9 promotes pancreatitis and pancreatic cancer in mice. Science.

[161] Freidlin B., Korn E.L., Gray R., Martin A. (2008). Multi-arm clinical trials of new agents: Some design considerations. Clin. Cancer Res..

[162] Rodon J., Soria J.C., Berger R., Miller W.H., Rubin E., Kugel A., Tsimberidou A., Saintigny P., Ackerstein A., Brana I. (2019). Genomic and transcriptomic profiling expands precision cancer medicine: The WINTHER trial. Nat. Med..

[163] Perera-Bel J., Hutter B., Heining C., Bleckmann A., Frohlich M., Frohling S., Glimm H., Brors B., Beissbarth T. (2018). From somatic variants towards precision oncology: Evidence-driven reporting of treatment options in molecular tumor boards. Genome Med..

